# Refined distributed emotion vector representation for social media sentiment analysis

**DOI:** 10.1371/journal.pone.0223317

**Published:** 2019-10-24

**Authors:** Yung-Chun Chang, Wen-Chao Yeh, Yan-Chun Hsing, Chen-Ann Wang

**Affiliations:** 1 Graduate Institute of Data Science, Taipei Medical University, Taipei, Taiwan; 2 Clinical Big Data Research Center, Taipei Medical University Hospital, Taipei, Taiwan; 3 Institute of Information Systems and Applications, National Tsing Hua University, Hsinchu, Taiwan; Politechnika Krakowska im Tadeusza Kosciuszki, POLAND

## Abstract

As user-generated content increasingly proliferates through social networking sites, our lives are bombarded with ever more information, which has in turn has inspired the rapid evolution of new technologies and tools to process these vast amounts of data. Semantic and sentiment analysis of these social multimedia have become key research topics in many areas in society, e.g., in shopping malls to help policymakers predict market trends and discover potential customers. In this light, this study proposes a novel method to analyze the emotional aspects of Chinese vocabulary and then to assess the mass comments of the movie reviews. The experiment results show that our method 1. can improve the machine learning model by providing more refined emotional information to enhance the effectiveness of movie recommendation systems, and 2. performs significantly better than the other commonly used methods of emotional analysis.

## Introduction

Thanks to the booming development of social media in recent years, we can easily acquire vast amounts of user-generated data. Valuable and up-to-date information is an important source of knowledge that can help us understand the public perception of certain issues [1, 2]. However, a substantial amount of information is exaggerated and false, which has prevented us from efficiently utilizing the wealth of information from social media. For this reason, in recent years, natural language processing (NLP) technology has been widely applied to social media data analysis. In particular, research on opinion and sentiment analysis has been widely adopted by businesses. They collect social media information from multiple sources as a means to determine the trends of public opinion so that they respond quickly and create competitive advantage [1]. This indicates that sentiment and opinion analysis of social multimedia has become a crucial step in understanding social trends and phenomena. As a result, sentiment analysis and emotion recognition have attracted much attention recently.

Sentiment classification, which aims at classifying sentiment data into polarity categories (e.g., positive or negative), is widely studied because many users do not explicitly indicate their sentiment polarity, thus we need to predict it from the text data generated by users. For instance, a sentiment classifier might classify a user review about a movie as *positive* or *negative* depending on the sentiment expressed in the review. Existing sentiment analyses mostly rely on a sentiment lexicon to represent the text. However, as the domain of the text changes, the lexicon may be insufficient due to the sparsity of domain-specific documents, which greatly hinders the effectiveness of sentiment analysis models. In addition, with the change of time, many popular buzzwords of social media users, especially young people, are also changing with the times in response to different social phenomena or current events. Thus cross-domain sentiment classification algorithms are highly desirable to reduce domain dependency and manually labeling costs.

In light of these considerations, this work proposes a fine-grained sentiment analysis method based on Valence and Arousal prediction. We further utilize this method to imbue words with sentiment meanings and present a novel document representation model. We aim at capturing intricate sentiment semantics and the author’s feelings during the composition of a movie review. Combined with Support Vector Machines (SVM), we can effectively identify the sentiment behind the movie review. The proposed method achieved promising results on the DSA_W dataset for Chinese words from IALP 2016, which tied for first place with the best system. We demonstrate that the novel document representation can assist machine learning and deep learning models to better understand more sophisticated sentiment information.

The rest of the paper is organized as follows. In the next section, we review related work. We then move on to describe the proposed method for multi-aspect sentiment analysis in Section 3, with its performance being evaluated and compared with other well-known methods in Section 4. Finally, we offer conclusions and directions for future work in Section 5.

## Literature review

Sentiment analysis and opinion mining involve classifying textual data into binary (positive and negative) or multiple classes depending on the semantics and sentiment information. This can be understood as inferring the author’s tendency, opinion, and attitude towards a certain topic [3]. Thus, we can, for example, identify public trends by performing these analyses on social multimedia. A well-known application of these methods is on political campaigns. Candidates can monitor the public opinion on social media and propose policies that are popular, so as to gain more support [4]. For example, during the election, the Mayor of Taipei, Dr. Ko, directed his campaign based on popular keywords and trend leaders that were uncovered by opinion mining; in the end, he won the election [5]. This is evidence that mining and analyzing social media can indeed help us understand—and take advantage of—public opinion and current trends. The importance of sentiment analysis in social media has therefore attracted considerable research interest. Turney et al. [6] attempted to search for adjectival or adverbial phrases in order to judge the sentiment polarity of movie reviews. They did so by calculating the correlation between these phrases and the words “excellent” and “poor”. Their research concluded that sentiment analysis of movie reviews is more difficult than product reviews, in that words describing the plot or actors can be easily mistaken as the sentiment of the author. For instance, if a review contains sentences like “the acting is bad and boring”, a sentiment analysis model may wrongly regard the words “bad” and “boring” as the author’s opinion towards the movie instead of the actor, therefore affecting the result of its analysis. A good example of this is with the movie “Pulp Fiction” (https://www.imdb.com/title/tt0110912/). It is a top ranked movie in the IMDB best movie list with a high score of 8.9/10 and one Oscar award. However, the content of most reviews is full of negative words that are used to describe movie plot but are not representative of the overall evaluation of this movie. This disconnect will mislead machine learning methods to make incorrect predictions. Pang et al. [4] investigated the problem of classifying documents by overall sentiment, and thus proposed a solution using a maximum entropy (ME) optimization process, the Naïve Bayes classifier and Support Vector Machines [7]. Their work first introduced these three machine learning methods to sentiment analysis and found that they outperformed human-produced baselines. Salton and Buckley [8] introduced a single-term-indexing model based on TF-IDF, a common word weighting mechanism in sentiment analysis and categorization models.

Since a large TF-IDF value indicates a higher correlation to a category, the model can compare with another more elaborate content analysis procedures.

Martineau and Finin [9] further improved this model and proposed the Delta TF-IDF, as shown in (1). Given a training dataset comprised of positive and negative documents, *V*_*t*,*d*_ is the feature value for term *t* in document *d*, *C*_*t*,*d*_ denotes the number of times term *t* occurs in document *d*, *P*_*t*_ represents the number of positive documents with term *t*, and |*P*| is the total number of positive documents. Similarly, *N*_*t*_ and | *N*_*t*_ | represent the number of negative documents with term *t*, and the total number of negative documents, respectively. They evaluated the performance of the classifier on English movie reviews and showed that Delta TF-IDF can effectively improve the sentiment analysis model.

$$\begin{array}{ll} V_{t,d} & =C_{t,d}*log_{2}\left(\frac{\left|P\right|}{P_{t}}\right)-C_{t,d}*log_{2}\left(\frac{\left|N\right|}{N_{t}}\right)\\ & =C_{t,d}*log_{2}\left(\frac{\left|P\right|N_{t}}{P_{t}\left|N\right|}\right)\\ & =C_{t,d}*log_{2}\left(\frac{N_{t}}{P_{t}}\right) \end{array}$$ (1)

**Fig 1:**
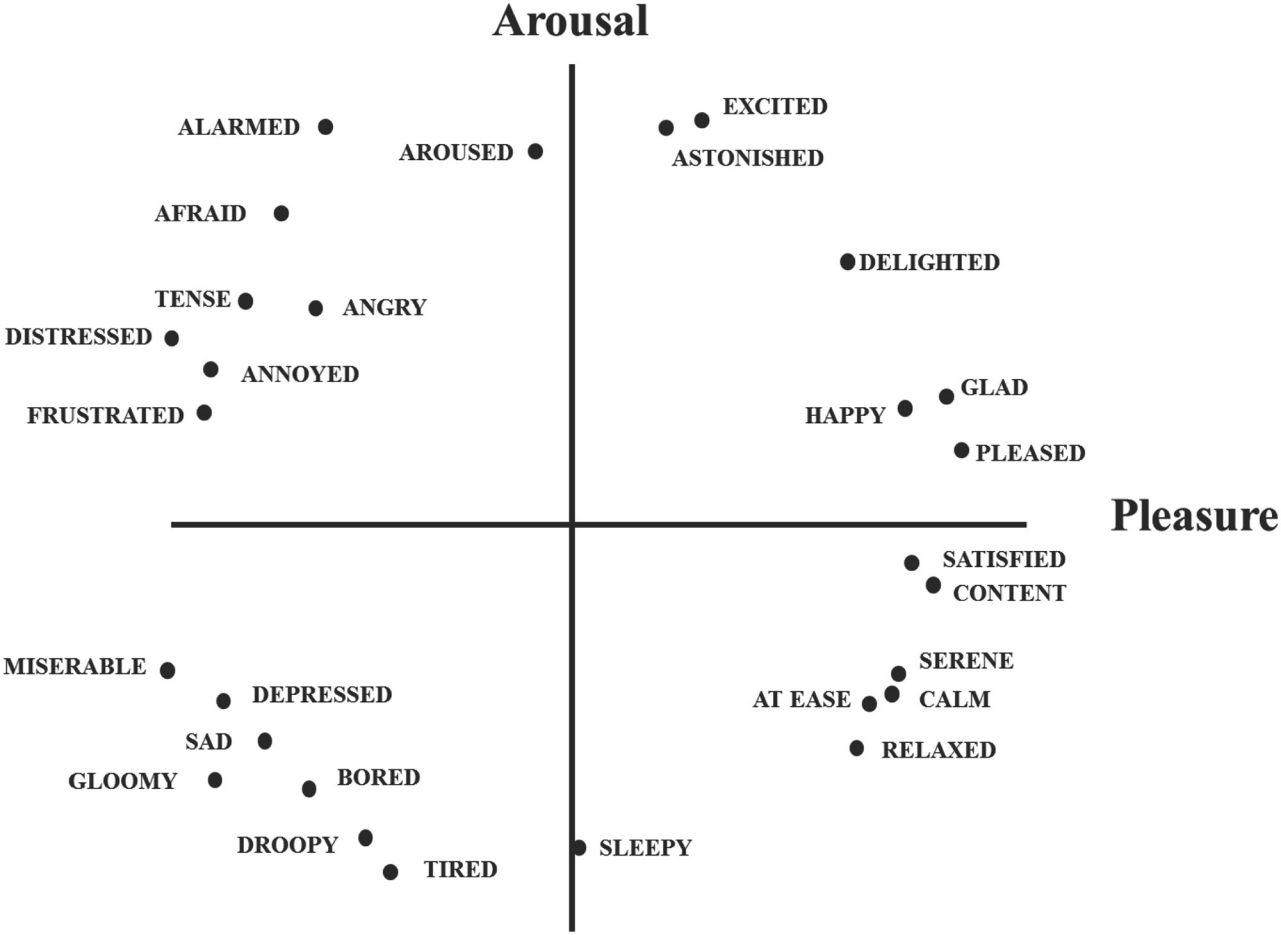
Distribution of 28 emotions in the circumplex model [13].

In recent years, there has been a dichotomy in the approach to defining emotion, from either Discrete Emotions Theory or the Dimensional Theory of Emotion. Gray and Watson [10] adopted the former theory which argues that emotion is innate, with different emotions all present at birth and physically distinguishable through the human body and other anatomical features. Plutchik et al. [11] drew on the discrete theory and further claimed that emotions can be categorized by different functions in various scenarios. On the other hand, the Dimensional Theory regards emotions as a distribution on one or several planes instead of being discrete. Although the term “dimension” is most commonly seen in mathematical or physical, mechanical systems, Osgood et al. [12] proposed its application to semantic analysis. Their work argued that most semantic differences can be attributed to three basic dimensions, i.e., “pleasure”, “arousal”, and “dominance”. Russell et al. [13] incoporated this theory into sentiment analysis and found that it could also be used to distinguish emotions. The distribution of emotions appeared to be circular with regards to the center of the axes. They further proposed the Circumplex Model (CM) comprising of two axes, “pleasure” and “arousal”. As shown in Fig 1, 28 emotions can be distinguished using these two axes. In particular, “pleasure” represents the polarity of the emotion, and “arousal” represents the strength of it. In this model, emotions with a positive polarity include happy, pleased, satisfied, etc., while negative emotions contain depressed, afraid, and frustrated. Meanwhile, on the arousal axis, strong emotions include excited, astonished, alarmed, etc., while weaker emotions include tired, sleepy, and bored.

**Fig 2:**
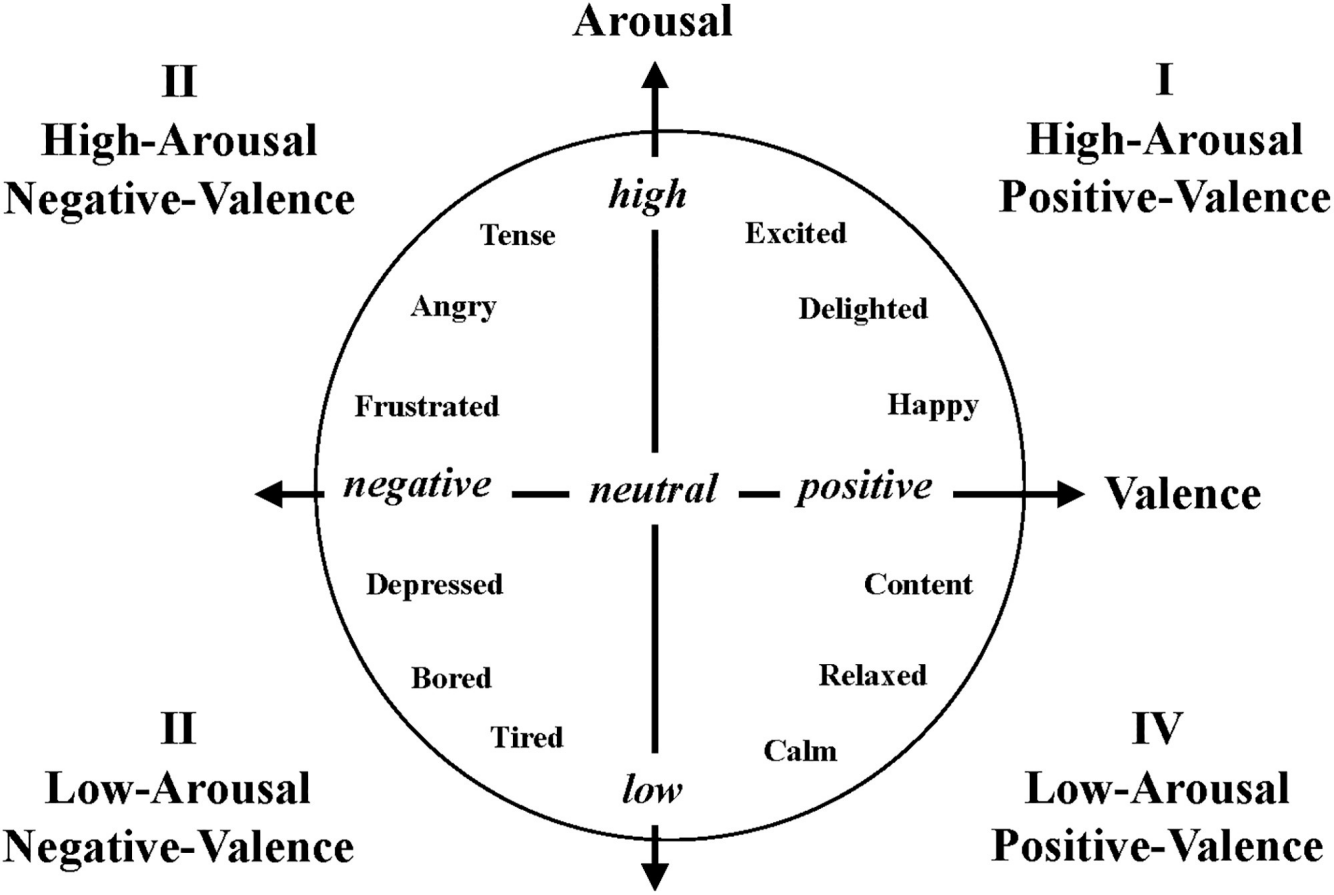
Emotions in the valence and arousal dimensions [14].

Existing sentiment analysis methods often utilize keyword extraction to determine the category of emotion and require the collection of a domain-specific emotion lexicon. For instance, the word “cry” is usually negative in our daily lives due to its link to negative emotions. However, in movie reviews, it can indicate that the movie is so touching that the viewer cried tears of compassion, thus transforming cry into a positive review for the movie. This demonstrates that domain-specific lexicon is unsuitable when the target of the analysis is changed. Thus, keyword-based models suffer from a lack of flexibility and a time-consuming data collection process. In 2015, Yu et al. [14] extended the CM to construct a Chinese emotional word model that can perform a more fine-grained analysis. In contrast to CM, they defined the vertical axis as “Valence”, and the range of both axes are constrained within 1–9, as shown in Fig 2. The meaning of Valence is the polarity of an emotion, where positive emotions include happy, delighted, and calm, and negative ones include depressed and frustrated. Arousal, on the other hand, indicates the degree of the emotions, where stronger emotions include angry and excited, and weaker ones include tired and calm. In this model, the emotion information of a word or sentence can be captured by its position in the valence-arousal plane [15]. In addition, Wang et al. [16] adopted E-HowNet to find synonymous words in the training data and used them to extend the training data in their model. This study further extends this method and combines character embeddings to construct Valence and Arousal inference models.

Social multimedia data from online platforms like Twitter and Facebook contain vast amounts of short texts (less than 200 words) in addition to normal news articles and videos [17]. Unfortunately, the length limitations in some social media can lead to the substantial omission of textual information. Context information may be therefore ignored, but this lack may hinder the performance of machine learning models in the task of understanding short texts. Research on short text representation has thus recently emerged as a popular topic. For example, Bharath Sriram et al. [18] first extracted domain-specific features from the text author’s CV and documents before using these features to classify the document as news, comments or personal information. In this way, they were able achieve improved performances over other short text classification methods.

Most of previous works on opinion analysis predominantly relied on the polarity of keywords to determine the opinion, and ignored the emotional meaning of other words [19]. However, small differences in the emotion of various words can greatly affect the overall opinion of the article. As such, we propose a finer emotional analysis model that incorporates not only the polarity but also the distribution of multi-dimensional emotion, in order to determine the global emotion category. Our novel method relies on the valence-arousal representation of emotions, and represents each word as a continuous, multi-dimensional vector. In this way, a classifier can better utilize the finer differences between emotional words. We conducted an experiment on movie reviews collected from online social multimedia and validated the proposed model as being better able to utilize the valence-arousal distribution achieve effective representations for subtle emotions. The experiment results show that this approach outperforms common sentiment analysis methods in identifying detailed emotions in a movie review. Furthermore, we visualize the extracted emotion information so as to exhibit the potential of the proposed method in unveiling finer public opinion towards certain topics and consequently understand the social trends and developments.

## Methodology

**Fig 3:**
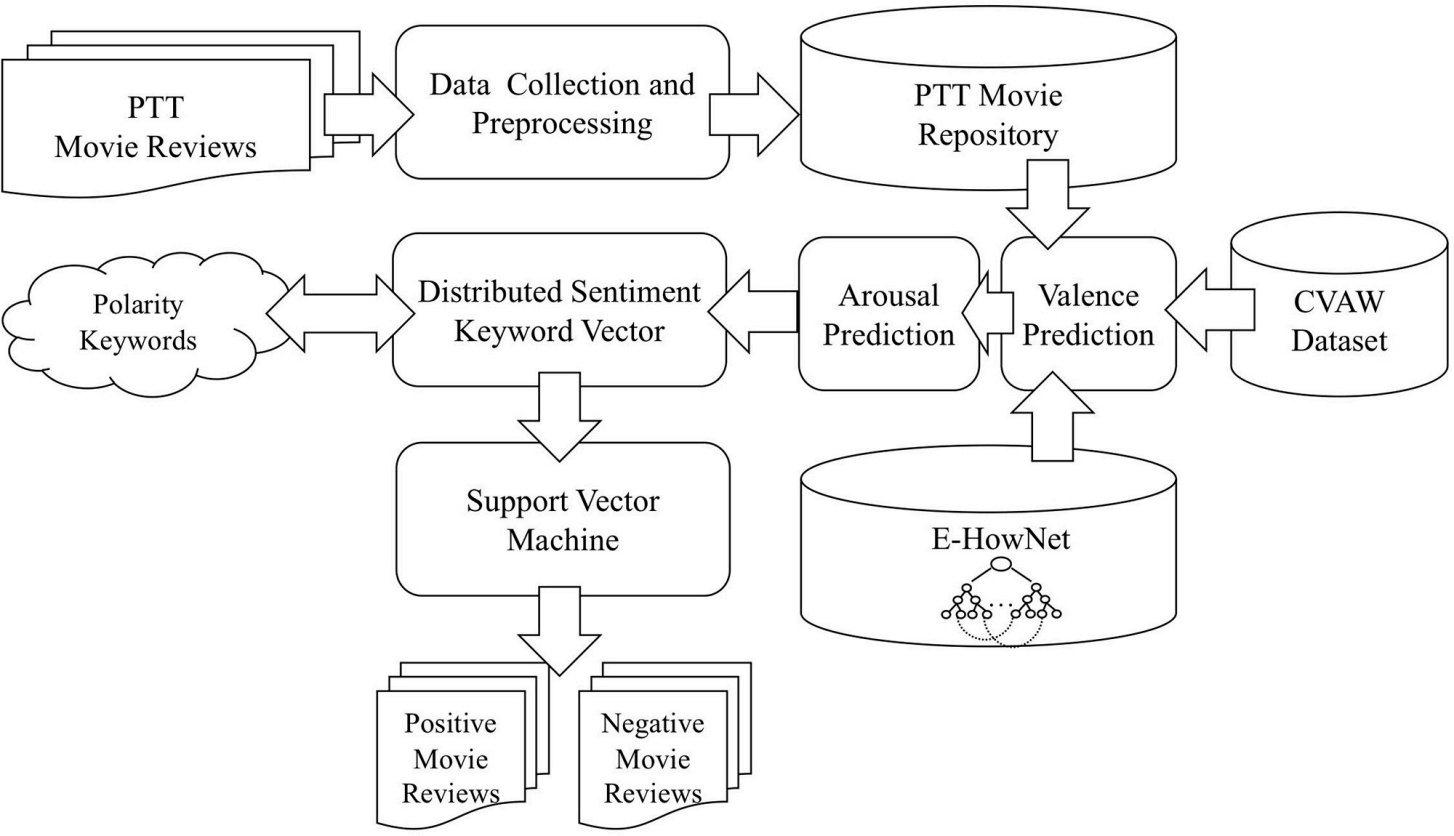
Movie review analysis framework based on valence-arousal representation model.

We propose a novel method for sentiment analysis that considers a word’s finer emotion connotation by combining the valence-arousal representation model and machine learning. This method is designed to examine movie reviews collected from social multimedia and efficiently extracts detailed emotion information. Fig 3 illustrates our classification framework. First, we collected a corpus of movie reviews from an online bulletin board (PTT, https://www.ptt.cc/bbs/index.html) as the target of our sentiment analysis, and we performed a preprocessing. Then, we utilized an external knowledge base to conduct the Valence and Arousal Prediction for every word. These predictions were subsequently combined with polarity keywords in the proposed Refined Distributed Emotion Vector (RDEV) representation method so that every review was projected onto a continuous, high-dimensional semantic space. Finally, we employed Support Vector Machines (SVM) to learn the classification model in order to identify the semantic category of the reviews. We explain the implementation details of each component in the following sections.

### Data collection and preprocessing

PTT is an electronic bulletin board system that provides a social platform for academic purposes. The system consists of numerous virtual “boards”, each with a different topic, so that a user can find and focus on one specific topic at a time. For example, the “Movie” board provides a space for users to discuss various aspects of movies. There is even a convention where the title of a post may contain the phrase “positive spoiler,” which indicates that this post contains spoilers and positive comments of a movie. Meanwhile, the phrase “negative spoiler” in the title suggests that the author of this post does not recommend this movie. Thus, we can conveniently construct our movie review corpus based on these key phrases. We collected 4,528 posts from 2004 to July, 2016, of which 2,264 are positive and the others negative.

To preprocess the corpus, we used MONPA (http://monpa.iis.sinica.edu.tw:9000/chunk) [20], a multi-objective annotation system based on bidirectional recurrent neural networks that simultaneously performs word segmentation, part-of-speech (POS) tagging, and named entity recognition. This process enables us to extract keywords more easily later. We then removed all stopwords (https://github.com/stopwords-iso/stopwords-zh) and proceeded with the following steps.

### Valence-arousal prediction

**Fig 4:**
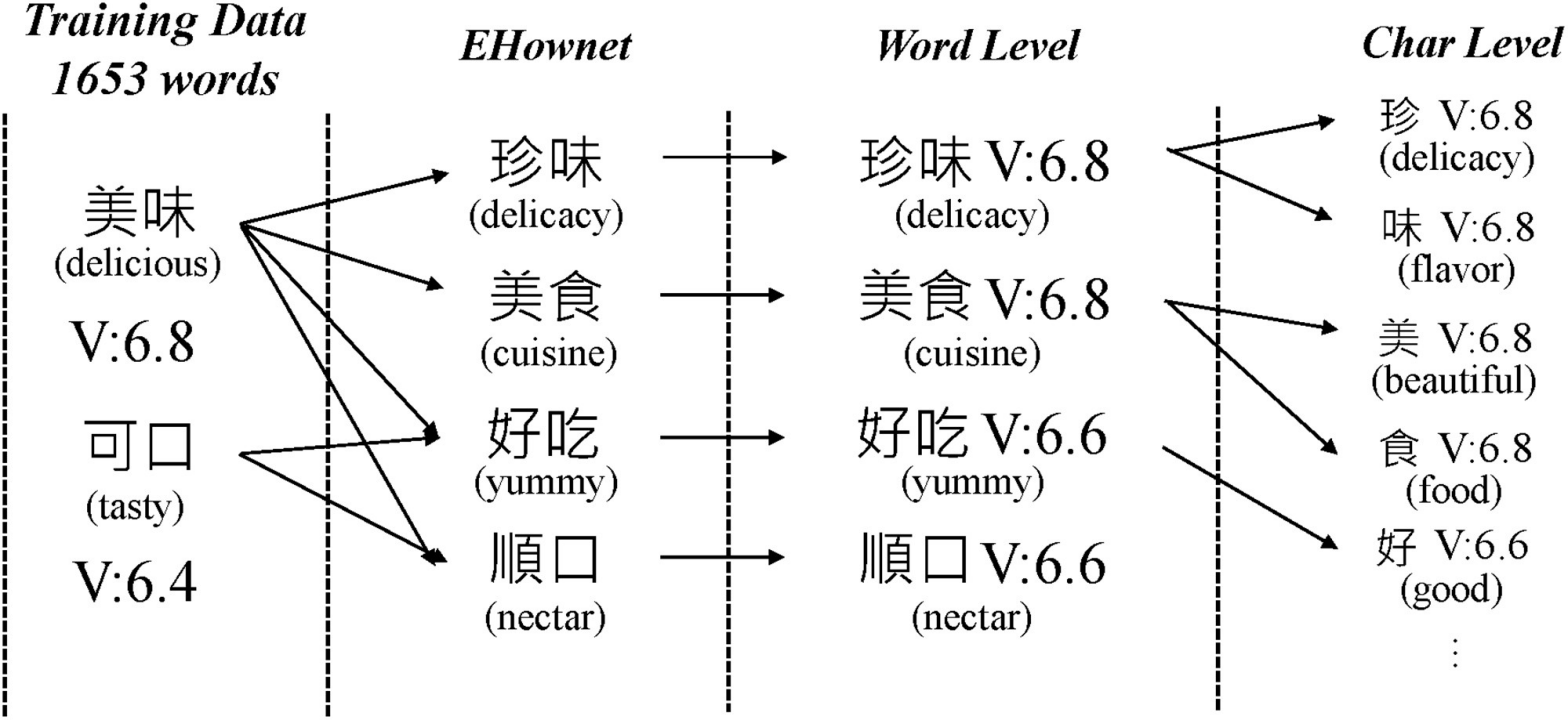
Valence extension using E-HowNet.

The Valence-Arousal Prediction module is based on the training data from the 2016 Chinese Valence-Arousal Words (CVAW, http://nlp.innobic.yzu.edu.tw/resources/cvaw1_download.html) 1.0 competition [21]. However, this data contains only 1,653 words and their valence-arousal value, which is insufficient for analyzing sentiments in social media. To overcome this limitation, we devised a method to extend this data following the assumption that synonyms of a sentiment word possess similar valence-arousal values. For instance, “happy” and “delighted” are synonyms, and their positions are close on the valence-arousal plane. Thus, we employed the E-HowNet (http://ehownet.iis.sinica.edu.tw/index.php) constructed by Academia Sinica to broaden the sentiment lexicon through a synonym lookup. Fig 4 exemplifies the valence value extension process of words “delicious” and “tasty”. Since E-HowNet represents words in a hierarchical sense structure, we can find words such as “delicacy” and “cuisine” in the same sense category, which are synonyms of the query word “tasty”. In this way, we can assign valence and arousal values to those words in our newly created corpus that are unseen in CVAW 1.0 by propagating the values among synonyms. Note that, if a word is synonymous to multiple words in CVAW, its value will be calculated as the average of those known words. Take the word “yummy” in Fig 4 as an example. Its valence will be the average of words “delicious” and “tasty”. In the end, we were able to obtain word-level valence-arousal (WVA) values of 13,578 words.

Although we substantially extended the sentiment lexicon to include the majority of everyday Chinese words and their corresponding WVA values, we can still find words in our corpus outside of our WVA wordlist. This is not surprising since typos and slang words are very common on social media.

In order to enhance our model’s ability to predict the sentiment of these unseen words, we further tokenized those words into individual characters. This is because Chinese words are usually composed of two or more characters, which contain a wealth of meaning information [22]. Moreover, Li et al. [23] also concluded that for many words and even phrases in Chinese, the semantics of their individual characters contribute strongly to the semantics of the whole. This especially holds when the component characters are synonyms or near synonyms. In this way, these characters can then inherit the WVA values of the words. For instance, the word 美味 (delicious) has a valence value of 6.8, so each character in this word will also get the same value. Obviously, some characters are shared among different words, so their value will be the average of all those words that contain this character. Finally, we obtained 3,748 character-based valence-arousal (CVA) values, and used them to estimate the the WVA of unseen, multi-character words in the corpus.

**Fig 5:**
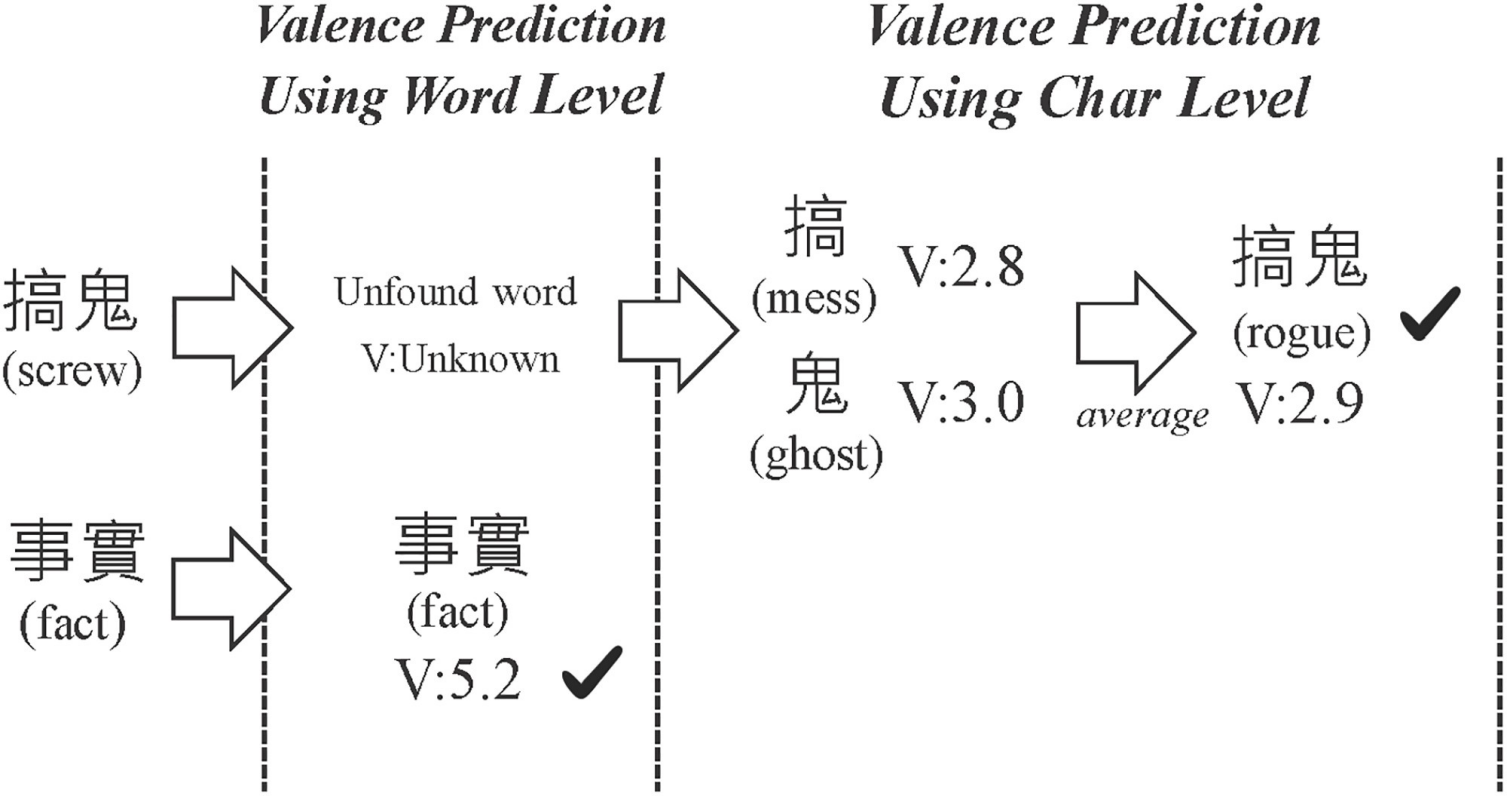
Word valence prediction based on character valence values.

Yu et al. [24] discovered that an ensemble of valence-arousal methods can improve the overall success of predicting the sentiment of a text. Thus, we further propose two combined approaches to predict valence values: 1) calculations using character-based valence, and 2) predictions based on k-NN. The two values from both methods will then be combined to obtain the final valence value. As shown in Fig 5, the word 事實 (fact) exists in the WVA data, so its valence value is known to be 5.2. However, the word 搞鬼 (screw) is unseen. So, as in the first approach, we obtained character valence values of each character, and then averaged them to obtain a valence of 2.9 for this word.

**Fig 6:**
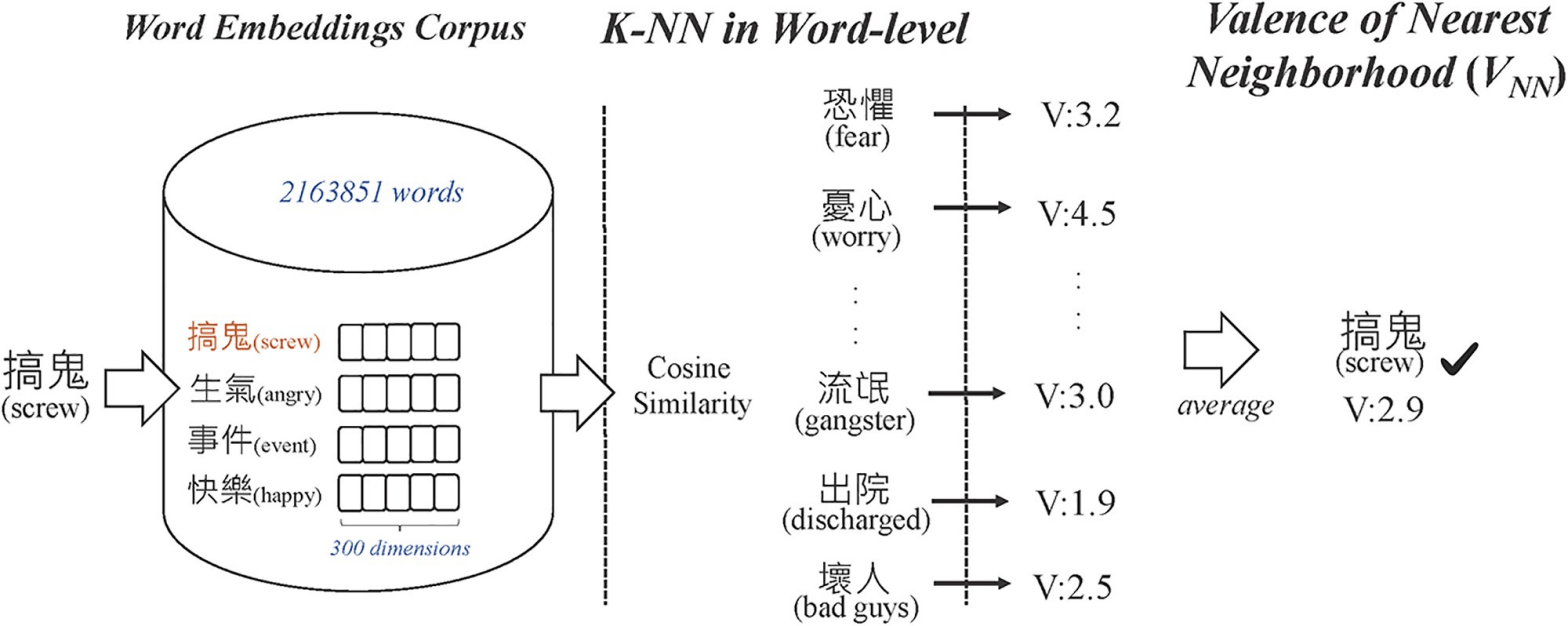
k-NN-based valence estimation.

On the other hand, our second approach, based on k-NN, was implemented as shown in Fig 6. Mikolov et al. [25] discovered that semantically similar words can cluster together in the word embedding space. This inspired us to find the valence of unknown words from their neighboring words. For example, the word “playground” is surrounded by others such as “happy”, “fun”, and “exciting”, in the word vector space. These words can be used to obtain an average of valence value of the target word “playground”. But before doing so, we need well-trained word and character embeddings. Therefore, we collected news and blog articles from the years 2008 to 2013 (approximately 3.6 million documents) as the training data for word embedding. Then, we employed word2vec (https://code.google.com/archive/p/word2vec/) to learn both word and character level embeddings. By doing so, we can assess word embeddings of unknown words by using their corresponding characters. Next, according to [26], the valence value can be predicted more efficiently when setting the number of nearest words to ten. We hence calculate cosine similarity between words in the vector space and then select top ten closest words with known valence values. Finally, the valence value of the unknown word can be estimated by a weighted average of the valence values of the neighboring words.

The valence values from the above two approaches will be further averaged to obtain the final prediction. We designed these approaches with the aim of preventing a strong bias from only one neighbor word. By taking an average of several neighbors, we alleviate the problem of a neighbor having an opposing valence tendency with the target word that we want to predict.

**Fig 7:**
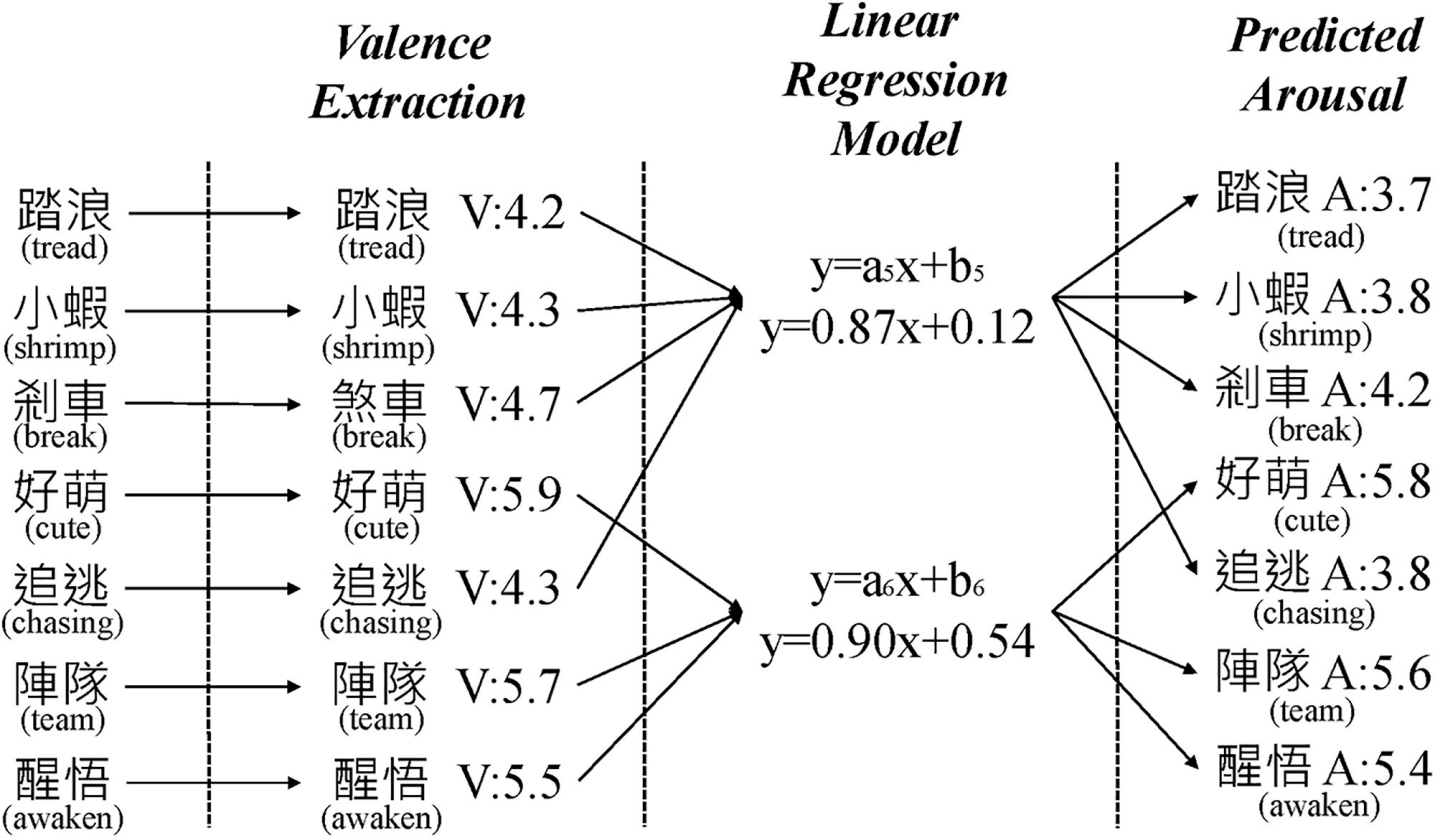
Linear regression-based arousal value prediction.

As for the prediction of the arousal value, we also put forward two approaches: 1) linear regression-based prediction, and 2) support vector regression-based prediction. The results of these predictions will be averaged, similar to the valence estimation process above. The first method is illustrated in Fig 7, where a linear regression model is trained to forecast the arousal value. We learn the linear regression function in order to project word-based valence to arousal values. This study assumes that the Valence value affects the Arousal value, so we use the valence-arousal values of the emotional facet vocabulary in WVA to learn the linear regression equations in each value field based on the 1–9 range of the emotional facet. In addition, we observe that valence values are not distributed evenly, with most of them falling into the scope of 3–7. As a result, data for extreme valence values are lacking. We therefore duplicate words that fall into these categories and assign them to adjacent groups. More specifically, let us consider the word “killed” with the valence value of 1.6. We will assign it to the groups 1 and 2. In this way, each and every group can have enough training data to learn a more accurate regression coefficient. Finally, we can predict arousal values for unknown words using a corresponding linear regression function of their group.

**Fig 8:**
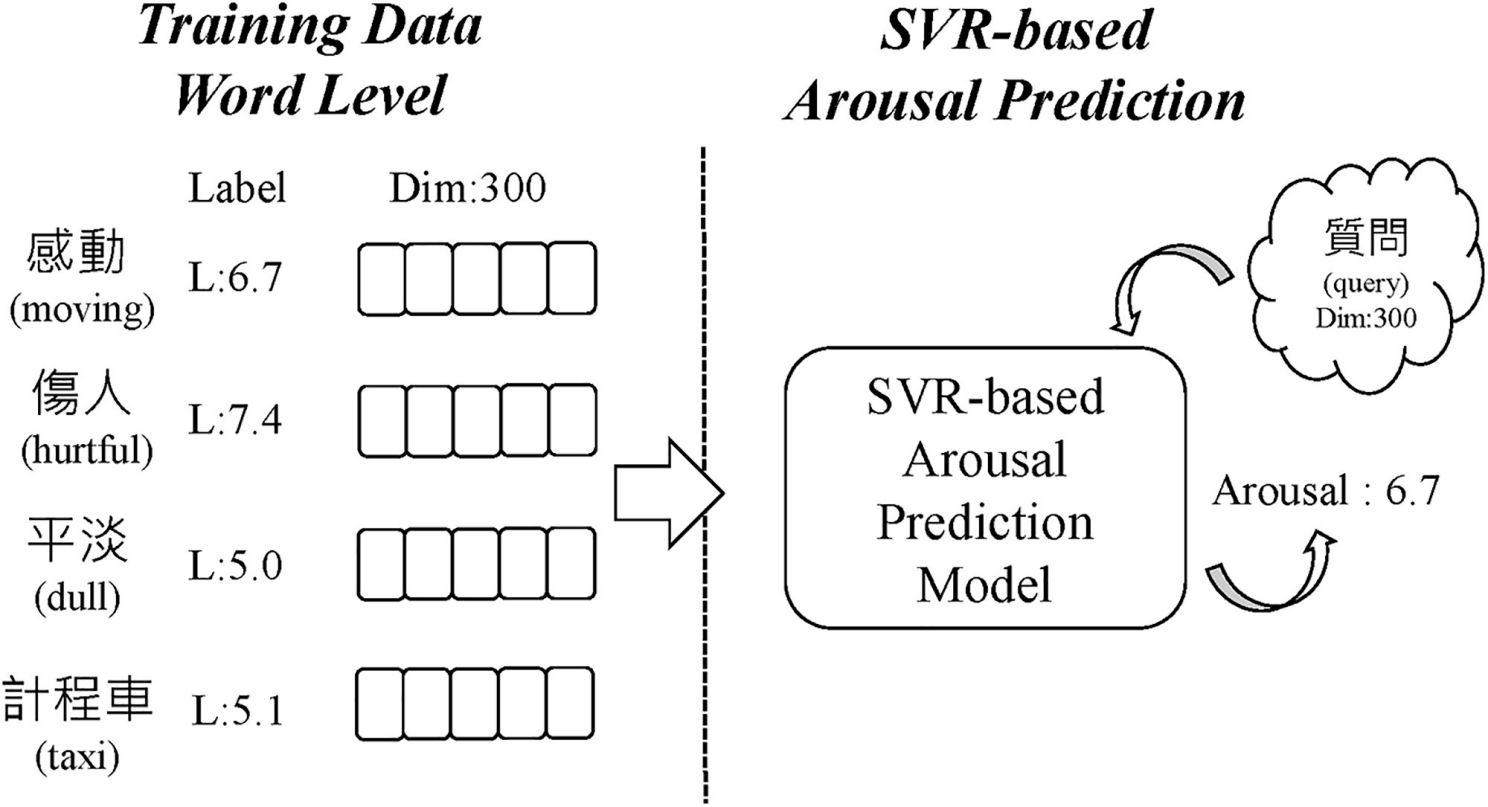
Support vector regression for arousal value estimation.

As for the second approach, namely, the support vector regression (SVR) approach, we first used word embeddings with 300 dimensions to represent the words. As shown in Fig 8, *L* denotes the value of the training data, *Dim* the dimension of the features, and an SVR model is trained to predict the arousal value. We subsequently combined the results from the two approaches to acquire the final arousal value. From our observations in this study, the values based on linear regression are more convergent, while the SVR values are more divergent. Therefore, the mean value of those two methods was used to improve their respective shortcomings and our ensemble model can arrive at a more balanced distribution of arousal scores that mitigate the shortcomings of both methods.

### Refine distributed emotion vectors representation

The proposed “Refined Distributed Emotion Vector (RDEV)” module combines the preprocessed text and their valence-arousal values so that more fine-grained emotion semantics can be well-represented. Traditional vector-based methods, such as the well-known Bag-of-Words (BoW), face with problems such as data and feature sparsity, which can lead to inaccuracy [27–28]. More recently, on the other hand, text representation has been significantly improved by neural network-based approaches. Since word embedding is able to capture the syntactic structure, context information, and semantics of a word in a document, it has thus become the most popular text representation approach [29–30]. Many NLP tasks have successfully benefited from word embedding, such as sentiment analysis [31], named entity recognition [32], and dialog systems [33].

**Fig 9:**
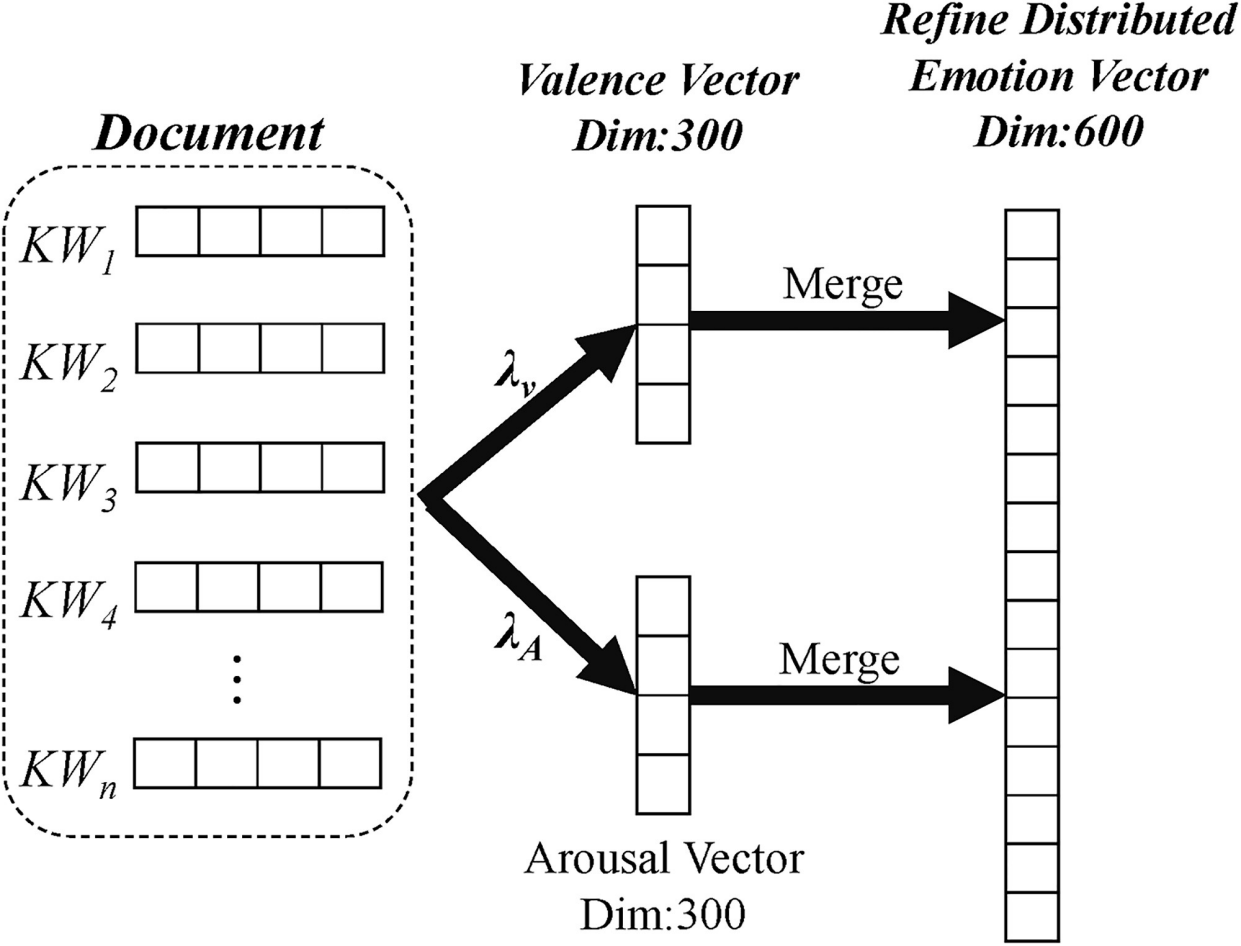
Refined distributed emotion vector construction process.

**Fig 10:**
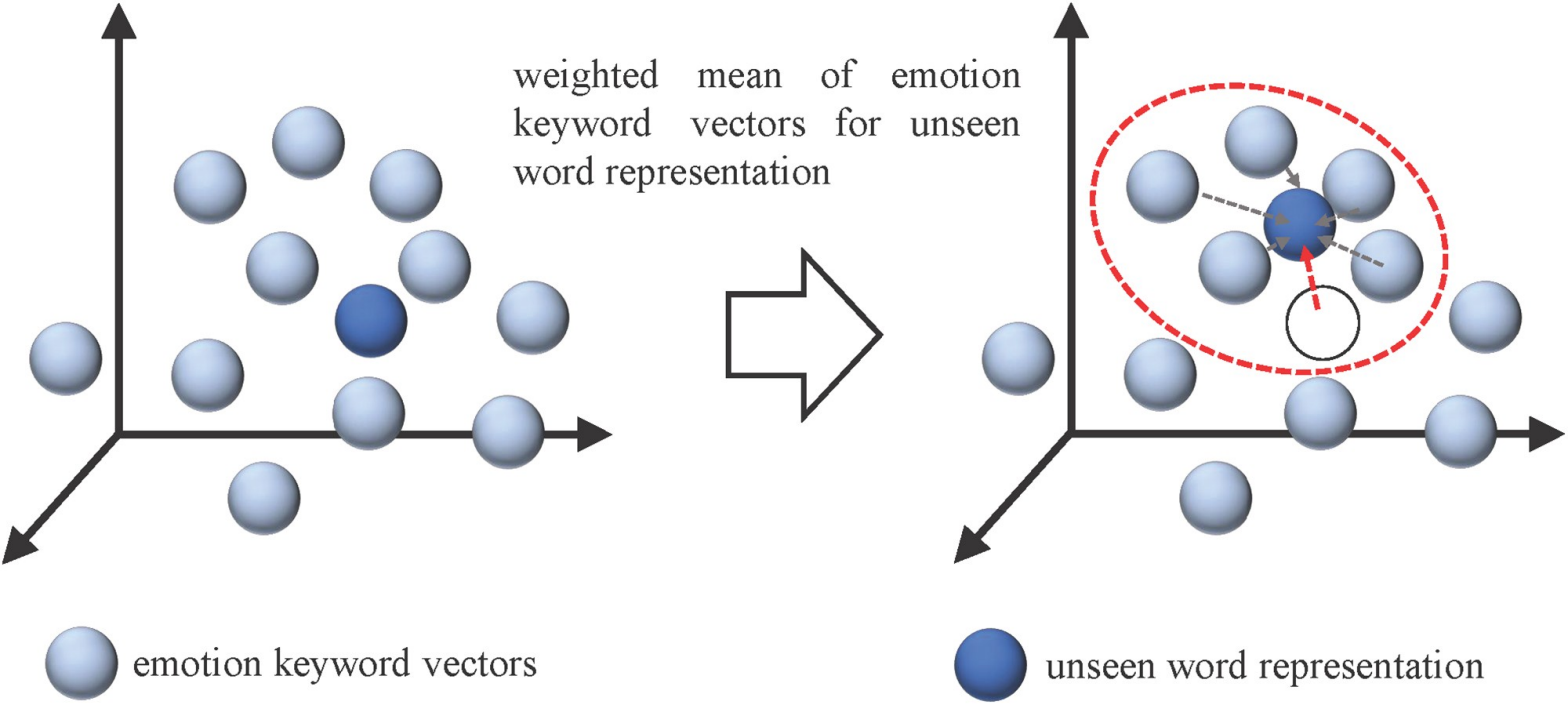
Estimation of a vector representation of an unknown word in RDEV.

To construct the RDVER for the text representation, we first utilized the continuous bag-of-word (CBOW) [25] model to learn word embeddings for the representation of text segments. The CBOW method learns word embeddings from a sequential word context. For the target word *w*_*t*_ at time step *t*, the CBOW model receives a window of *n* words around *w*_*t*_, and the loss function *J* can be written as (2):

$$J=\frac{1}{T}\sum_{t=1}^{T}logP\left(w_{t}|w_{t-n},\ldots ,w_{t-1},w_{t+1},\ldots ,w_{t+n}\right)$$ (2)

where *n* indicates the window size of the training context for the central word *w*_*t*_, and the vocabulary size of training corpus is denoted as *T*. Once we obtain the embeddings of each word from the corpus, the RDEV calculates a dense vector representation of the text by considering all of its word embeddings. Therefore, it leverages the advantages of combining semantics in a vector space through simple arithmetic. Given a movie review *R*_*t*_, we retrieved word embeddings with 300 dimensions of words *W* = {*w*_*1*_, *w*_*2*_, …, *w*_*n*_} in *R*_*t*_, and λV, λA denote valence and arousal values of *W*, respectively. We then employed the weighted-average to integrate word embeddings with their respective valence and arousal values to produce refined distributed emotion embedding for text representation of the movie reviews in the training corpus. As shown in Fig 9, we computed vectors for valence and arousal separately, each with 300 dimensions, and then we concatenated them to form a more comprehensive representation of the sentiment information in the document. It is worth noting that Fig 10 exemplifies the process of finding an integrated vector representation of a new document. Thus, even if words in a document are unseen, we can locate the nearest five words through k-NN and use their valence-arousal values to construct RDEV.

In essence, each movie review is projected onto a point in the latent feature space as a distributed representation which can then be evaluated using any classifier. In comparison to the Bag-of-Words (BOW) model, this distributed model of movie reviews can incorporate a broader amount of context information that covers the entire review in the representation. Moreover, the semantic relations of various surface words can also be captured from the vector space projection of these distributed representations. Such characteristics cannot be easily accomplished in a BOW-based approach, which consumes a significant amount of storage for a sizable n-gram dictionary. Finally, we employ Support Vector Machines (SVM) to learn a sentiment classification model of textual data from online social media.

## Experimentation

### Performance evaluation for the prediction of Chinese valence-arousal words

We first evaluated the performance of various methods on DSA_W (http://nlp.innobic.yzu.edu.tw/tasks/dsa_w/) competition data, which contain 1,653 training and 1,149 testing words. The metrics used were Mean Absolute Error (MAE) and Pearson Correlation Coefficient (PCC). MAE, the definition of which is listed in (3), is designed to reflect the mean difference between predicted and real values, with a smaller value being better. Here, *A*_*i*_ denotes the answer, and *P*_*i*_ the model’s prediction of valence and arousal values, and *n* the number of test words.

$$MAE=\frac{1}{n}\sum_{i=1}^{n}|A_{i}-P_{i}|$$ (3)

The PCC’s purpose is to reveal the correlation between predicted and real values. The PCC ranges from -1 to 1, and a value closer to 1 indicates that the two numbers are more correlated, whereas a PCC between 0 and 0.09 shows that there is no correlation, and the range 0.1–0.3 means a low correlation. Next, 0.3–0.5 displays a medium correlation, and a PCC value larger than 0.5 means that there is a strong correlation [34]. PCC is defined as *r* in (4), in which the symbol *P*_*i*_ denotes the model’s output of valence and arousal values, *A*_*i*_ denotes the answer, $\overset{-}{A}$ and $\overset{-}{P}$ are arithmetic means of the previous *A*_*i*_ and *P*_*i*_, respectively; *n* is the number of testing data, and σ denotes the standard deviation.

$$r=\frac{1}{n-1}\sum_{i=1}^{n}\left(\frac{A_{i}-\bar{A}}{\sigma_{A}}\right)\left(\frac{P_{i}-\bar{P}}{\sigma_{P}}\right)$$ (4)

We discuss the performances of valence and arousal separately in the following section. Table 1 lists the outcome of the valence prediction methods, where V_WVA_ denotes the use of only word-level valence-arousal (WVA) information, V_CVA_ the use of only character-level (CVA) information, V_WCVA_ the combination of the word- and character-levels, V_kNN_ the use of k-NN as valence prediction method, and V_WVAE_ the use of word embeddings to find the closest neighbors and taking the average. We can discover from these results that V_WCVA_ and V_WVAE_ are the two strongest models; for MAE, they are 0.527 and 0.508, respectively, and for PCC, they are 0.816 and 0.824, respectively, all of which indicate very high levels of correlation. Therefore, we further combined these two models in the V_mixed_ model, which obtains 0.496 and 0.845 for MAE and PCC, respectively. This model is the best among all of our proposed ones.

**Table 1:** Evaluation of valence prediction methods.

	*V*_*WVA*_	*V*_*CVA*_	*V*_*WCVA*_	*V*_*kNN*_	*V*_*WVAE*_	*V*_*mixed*_
*MAE*_*V*_	0.701	0.616	0.527	0.778	0.508	**0.496**
*PCC*_*V*_	0.831	0.795	0.816	0.728	0.824	**0.845**

Furthermore, in a DSA_W valence prediction task, we compared our best method with the top three competitors, namely, Aicyber, ECNUCS, and CKIP. As listed in Table 2, our method achieves the lowest MAE among all compared teams, which indicates that it is better at approximating the true valence values. Although the team CKIP obtained the best PCC of 0.862, we still produced a comparably high performance of 0.845, which still shows a very high correlation between the outcome of our method and the correct valence values. We observed that teams CKIP and Aicyber used word embeddings from approximately 500,000 words, which enables them to more precisely search for synonyms that are semantically similar to the target word, which in turn improves the PCC metric. In addition, teams Aicyber and ECNUCS both utilized artificial neural networks for valence prediction, the benefits of which include iterative optimization to reduce the number of features; however, its drawbacks are the requirement of substantial computing hardware and time costs. In contrast, we designed our method with special attention to the conservation of resources and computation time. We consider this to be a more desirable quality especially when analyzing real-time online social media.

**Table 2:** Performances of valence prediction models in DSA_W competition.

	*CKIP*	*ECNUCS*	*Aicyber*	*Our Method*
*MAE*_*V*_	0.583	0.577	0.577	**0.496**
*PCC*_*V*_	**0.862**	0.811	0.848	0.845

As for the prediction of arousal values, Table 3 lists the performances of the various methods: R_Polyfit_ and R_Linear_, which respectively represent using Polyfit Regression and Linear Regression to predict arousal; R_WVA_, which is a method that uses WVA for linear regression; and finally, S_CVA_ and S_WVA_, which predict arousal respectively using WVA and CVA as features for SVR. The results show that R_WVA_ can achieve the best MAE score of 0.939, but its PCC is the worst. On the other hand, S_WVA_ has a slightly inferior MAE of 1.003 but attains a very strong PCC of 0.427. More importantly, we observe that the predictions of S_WVA_ has an even distribution very similar to the correct answers. Therefore, we further combined the regression-based method and SVR-based method to arrive at the best performing method, RS, which obtains a MAE of 0.858 and PCC of 0.474.

**Table 3:** Comparison of arousal value prediction methods.

	*R*_*Polyfit*_	*R*_*Linear*_	*R*_*WVA*_	*S*_*CVA*_	*S*_*WVA*_	*RS*
*MAE*_*A*_	1.043	0.953	0.939	1.281	1.003	**0.858**
*PCC*_*A*_	0.294	0.296	-0.003	0.367	0.471	**0.474**

Finally, we compare our best method with the top three competitors in DSA_W arousal prediction task, namely, YUN-ISE-HPC, NCYU, and KUAS-IsLab (Table 4). Here, we observe that our method has the lowest MAE in predicting arousal values in this dataset, which again shows that it can closely track the arousal values of the words. Yet we only have a comparable performance on the PCC of 0.474 to the best team’s 0.544.

**Table 4:** Performance comparison of our method and top teams in the arousal value prediction task in DSA_W.

	*YUN-ISE-HPC*	*NCYU*	*KUAS-IsLab*	*Our Method*
*MAE*_*A*_	1.084	0.992	0.953	**0.858**
*PCC*_*A*_	**0.544**	0.393	0.295	*0*.*474*

We posit that since team KUAS-IsLab relied solely on linear regression to predict arousal values, the distribution falls between 4 and 6. Thus, the PCC for their method is less than satisfactory. Team NCYU employed a similar approach for arousal prediction to their valence component, which ignored the difference between these facets. We posit that this may be the weakness in their method since there can be large distinctions between the polarity and degree of emotions in various words. Team YUN-ISE-HPC utilized two sets of embeddings (word2vec (https://code.google.com/archive/p/word2vec/) and GloVe (https://nlp.stanford.edu/projects/glove/)) for the representation of words and trained two models from respective embeddings before averaging them. This approach effectively lowered the error and boosted the correlation. We also believe GloVe embeddings to be better at comprehending the semantics of sentiments than word2vec, which contributes to the higher PCC value.

It is worth noting that, although the performance of our method for predicting valence-arousal is lower than the best system, the proposed method can achieve state-of-the-art performance on MAE. In order to evaluate the system performance with the combination of MAE and PCC, we referred to the DSA_W competition [21] to include the rank as the extra metrics, and adopted the mean rank for estimating overall performance. As shown in Table 5, our method tied with Aicyber for first place. These results suggest that the proposed method is superior to all other competitors at predicting the sentiment of Chinese words in terms of MAE, and it can obtain strong PCC as well.

**Table 5:** DSA_W Valence and arousal prediction task results.

System	Valence			Arousal			Overall
	*MAE* *(rank)*	*PCC* *(rank)*	*Mean Rank*	*MAE* *(rank)*	*PCC* *(rank)*	*Mean Rank*	*Rank*
ECNUCS	0.577(2)	0.811(5)	3.5	1.33(7)	0.617(3)	5	4.25
YUN-ISE-HPC	0.814(6)	0.766(6)	6	1.084(4)	0.544(4)	4	5
CKIP	0.583(4)	**0.862(1)**	2.5	1.303(6)	0.63(2)	4	3.25
NCYU	0.82(7)	0.615(7)	7	0.992(3)	0.393(6)	4.5	11.5
Aicyber	0.577(2)	0.848(2)	**2**	1.212(5)	**0.671(1)**	**3**	**2.5**
KUAS-IsLab	0.583(4)	0.817(4)	4	0.953(2)	0.295(7)	4.5	4.25
Our Method	**0.496(1)**	0.845(3)	**2**	**0.858(1)**	0.474(5)	**3**	**2.5**

#### Performance evaluation of sentiment analysis on Chinese movie reviews

The purpose of this experiment was to examine the effectiveness of the RDEV method in capturing sentiment information and representing it for a sentiment classification model for movie reviews from PTT. We used 4,528 posts (dating from 2004 to July, 2016) on the “Movie” discussion board, of which 2,264 are positive and the remaining half negative. We performed a 10-fold cross validation, and the metric for evaluation was accuracy. We compared the RDEV method with several other approaches including Naïve Bayes (NB), *k* Nearest Neighbors (k-NN), Bag-of-words SVM (SVM), Vector Space Model (VSM), a strong method for document sentiment classification, Delta TF-IDF (DT) [15], and a gradient boosting decision tree that integrates multiple learners for classification problems (XGBoost) [35]. Four well-known deep learning-based text classification approaches were also included: convolutional neural network for text classification (TextCNN) [36], recurrent neural network with long short-term memory (LSTM) [37], bi-directional LSTM (BiLSTM) [38], and recurrent convolutional neural networks for text classification (RCNN) [39]. Our method, SVM_RDEV_, utilized word2vec to learn word and character embeddings from 3.6 million news and blog texts. The toolkit *libsvm* (https://www.csie.ntu.edu.tw/~cjlin/libsvm/) was used to learn the SVM classifier.

For the parameter setting, all machine learning-based methods used for comparison were optimized by means of a unified parameter setting procedure against the evaluated dataset. The SVM classification has a regularization parameter *C* which prevents overfitting of classifier training. In this comparison, *C* was set at 0.125 and 0.25 for SVM and SVM_RDEV_, respectively. For the *k* Nearest Neighbors classifier, we set *k* as 5 after searching from 3 to 10. Note that the training and testing of the Naïve Bayes, Vector Space Model, and Delta TF-IDF text classification methods were based on term frequencies only and thus had no parameter tuning. For the XGBoost, the learning rate was 0.3, which was the step size shrinkage used in the update to prevent overfitting. The maximum depth of a tree was 6 and the tree construction algorithm was set up to use the exact greedy algorithm to choose the fastest method.

For the TextCNN method, the input layer was a 300-dimension word embedding. Next, we set the parameters for feature selection architecture at 2 filters with a region size of 5. 1D-max pooling was performed over each feature to capture the maximum value as the feature corresponding to the filter. Finally, a softmax layer received the feature vector input and used it to model classifiers for the movie review. Similarly, the input layer and output layer of LSTM was identical to TextCNN, which are the word embeddings layer and softmax layer, respectively. In the middle layer, an LSTM layer was adopted to extract features and serve as the input of the penultimate layer, which is the dense layer. Since binary classification is assumed, we depicted two possible output states (i.e., positive or negative). Moreover, the architecture of BiLSTM is identical to that of LSTM, with the only difference being that the LSTM layer is replaced with a Bi-directional recurrent neural network. The structure of RCNN is similar to that of TextCNN. We substituted the convolution layer of BiLSTM to learn the context information. Afterward, a max-pooling layer was adopted to further filter the features. Finally, a dense layer was employed for classification.

All DNN-based models were implemented using Keras (https://keras.io/). To obtain the word embedding for each of the tokens, we used Gensim word2vec (https://radimrehurek.com/gensim/models/word2vec.html) to learn word embeddings from the training dataset, with each token representing the use of a dense vector of size 300. In order to prevent over-fitting, we employed a dropout size of 0.25 on the convolution layers, max-pooling layers, LSTM layer, and fully connected layers. ReLU activation was used in Dense Layers to reduce the input tensor for binary classification output representation. The training was continued for at most 20 epochs.

Note that we only report the accuracy because the data distribution of positive and negative reviews were identical, which results in the precision, recall, F_1_-score, and accuracy being the same. As shown in Table 6, the k-NN method found three movie reviews that were the most similar to the current one by exploiting word embeddings and the *k*-nearest neighbor algorithm. Then it summed up the number of positive and negative words therein to determine the polarity of the current review. This straightforward method could only reach the lowest accuracy of 39.7%, due to the sparsity of training data that prevents the discovery of reliable clusters. Another strong baseline for the majority of text classification problems, NB, was found to have a substantial accuracy of 62.4%, which is only slightly inferior to the bag-of-words SVM method’s 69.3%. NB is based on the product of the probabilities of words being related to a certain category, whereas SVM lists the occurrences of words as features for the SVM classifier. We recognize in our data that the entire review contains the comments from other people in addition to the authors, and those comments often share a similar vocabulary. Therefore, a frequency-based model such as SB can more accurately predict the sentiment than the unsophisticated NB method. Next, we turn to the popular classification methods VSM and DT, which are very effective at the classification problems in English corpora. The accuracies of VSM and DT were 69.1% and 77.1%, respectively. This considerable difference can be attributed to the fact that DT considers the correlation between a word and all categories simultaneously. That is, if a word exists in more than one category, its weight will be tuned down so as to alleviate the risk of misclassification. Thus, DT is more successful at detecting the sentiment than VSM. It is worth mentioning that XGBoost outperforms most compared methods which can achieve approximately 82% accuracy. This is because XGBoost uses gradient boosted learning to train the model from relevant instances. As for the two basic neural network-based methods, TextCNN and LSTM, we can see that they both achieved an accuracy about 80%, which marks an improvement over traditional methods by roughly 3%. On the other hand, the advanced DNN approaches of BiLSTM and RCNN can further boost the performance to approximately 83%. This reveals that using bi-directional RNN to learn context information is efficient in opinion classification.

**Table 6:** Performances of sentiment analysis models on PTT movie reviews.

Term Frequency-based Method		Deep Learning-based Method		Machine Learning-based Method	
*method*	*Accuracy (%)*	*method*	*Accuracy (%)*	*method*	*Accuracy (%)*
k-NN	39.7	TextCNN	79.7	XGBoost	81.4
NB	62.4	LSTM	79.8	SVM	69.3
VSM	69.1	BiLSTM	82.3	SVM_RDEV_	87.8
DT	77.1	RCNN	81.1		

Last but not least, the proposed method SVM_RDEV_ surpassed all other approaches on this task with an overwhelming 87.8% accuracy. We believe this demonstrates that the proposed method, which considers both valence value as the polarity and arousal value as the strength of sentiment, can greatly improve the effectiveness of sentiment analysis. In addition, our model incorporated finer linguistic and semantic detail into the feature for the SVM classifier, which has also proven to be very crucial in correctly judging the sentiment of movie reviews.

In order to examine the effectiveness of RDEV in the fine-grained sentiment analysis task, we conducted an experiment of emotion recognition. We adopted the task of corpus emotion analysis in Chinese Weibo posts in NLPCC 2014 (http://tcci.ccf.org.cn/conference/2014/dldoc/evtestdata1.zip); here, each post is given an emotion tag from eight categories: *anger*, *disgust*, *happiness*, *like*, *sadness*, *fear*, *surprise*, and *none*. We ignored “*none*” since it is not considered an emotion category. In the end, a total of 19,389 posts were kept and divided into a training set and a test set according to the definition of NLPCC, each containing 15,592 and 3,797 articles (posts), respectively. The detailed data distribution is shown in Table 7. The evaluation metrics were precision, recall, and F_1_-score [40], as well as the weighted-average used for comparing the average performance. The F_1_ value was used to determine the relative effectiveness of the compared methods.

**Table 7:** Distribution of training data and testing data from NLPCC.

*Dataset*	Category	#Train	#Test	Total
***NLPCC*** ***(19389)***	Anger	1885	243	2128
	Disgust	3124	674	3798
	Happiness	2765	630	3395
	Like	4253	1629	5882
	Sadness	2457	299	2756
	Fear	299	67	366
	Surprise	809	255	1064

Table 8 displays a comprehensive performance evaluation of the proposed method and other methods. The first is a TF-IDF term weighting-based bag-of-word model which is trained by SVM (SVM). Another is a convolutional neural network model which represents a text as a feature map for recognizing emotion (TextCNN). The last is a recurrent neural network with long short-term memory for the emotion classification model (LSTM). We also included the results of the Naive Bayes (NB) and Logistic Regression (LR) classifier as a baseline. In order to examine the effectiveness of our proposed text representation method, we also compared the different methods which utilized the RDEV as text representation (i.e., SVM_RDEV_, TextCNN_RDE_V, and LSTMRDEV).

**Table 8:** Performances of sentiment analysis models on NLPCC dataset.

*Method*	*Performance (%)*		
	Precision	Recall	F_1_-score
NB	63.82	61.90	58.84
LR	57.80	56.99	56.53
SVM	63.86	63.49	59.85
TextCNN	68.50	67.37	67.62
LSTM	69.48	72.29	70.48
SVM_RDEV_	69.05	75.03	70.16
TextCNN_RDEV_	70.36	73.20	71.10
LSTM_RDEV_	71.10	75.60	72.39

The F_1_ scores of NB, LR, and SVM are generally worse than those of the compared deep learning-based methods. This is because these three approaches are based on TF-IDF term weighting, and thus neglect the context and sentiment information of the sentence that the other methods draw upon. In contrast, TextCNN and LSTM take advantage of the word embeddings as the text representation method to learn the syntactic structure of an emotional sentence. Hence, they surpass SVM by about 10% in terms of the F_1_-score. It is worth noting that the results demonstrate that the methods adopting our proposed RDEV representation method are able to enhance their classification performances, which can achieve more than 72% F_1_-score. The reason is that the emotion in the text is usually conveyed by specific terms. The proposed method can capture the sentiment information behind the terms in a sentence by calculating valence-arousal. Since we further integrated the sentiment information into the vector for text representation, it can effectively recognize emotion.

To summarize, according to the above experiment results, the RDEV can improve the performance of SVM, TextCNN, and LSTM by providing more detailed emotional information to enhance the effectiveness of sentiment classification, and thus performs significantly better than the other commonly used methods of emotional analysis.

**Fig 11:**
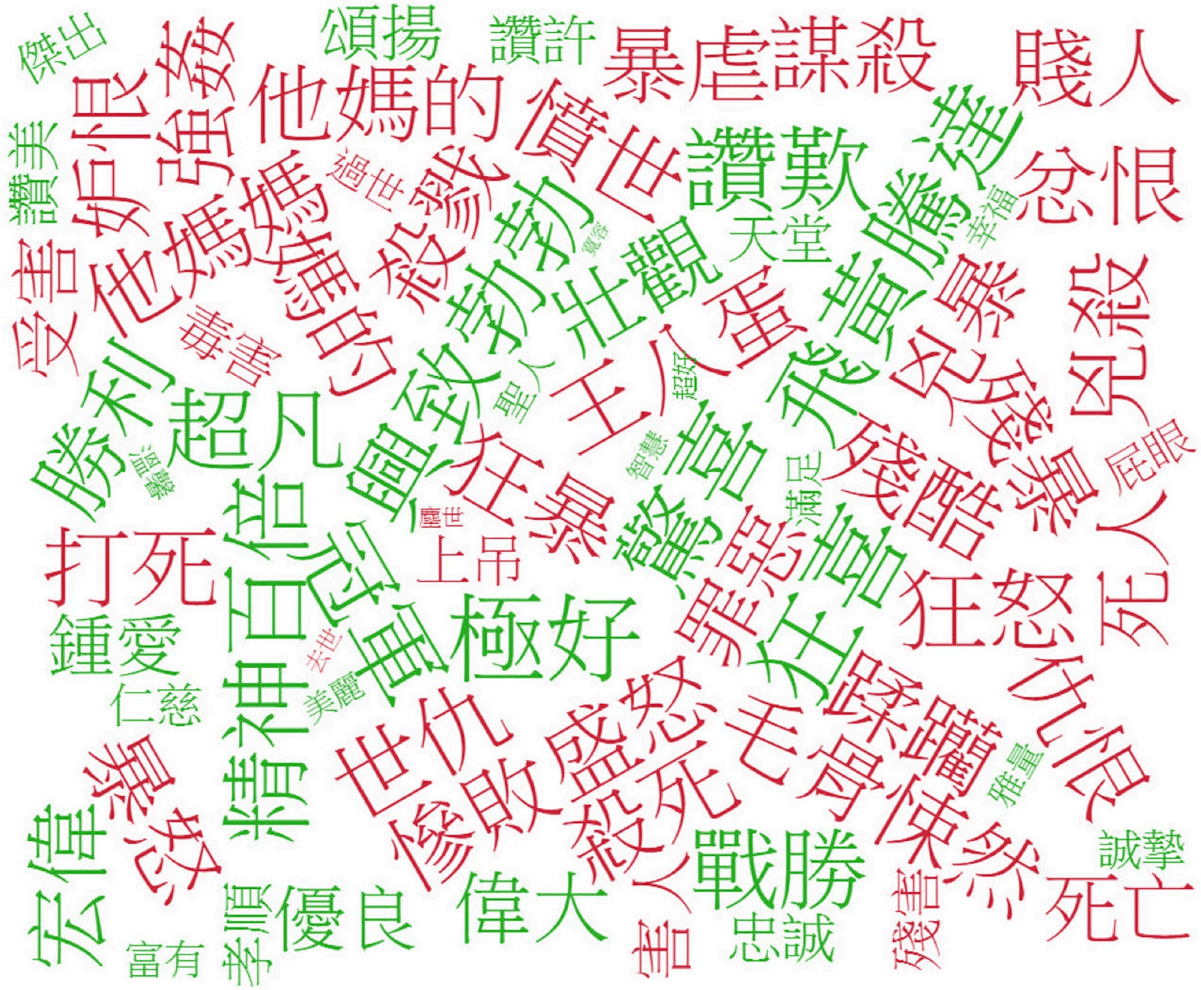
Word cloud from positive and negative movie reviews.

To gain a deeper understanding of reviews, we use word clouds to visualize the sentiment keywords. In doing so, specific sentiments can be clearly associated with their descriptions. To construct the word cloud, we select words with top the 100 valence values for the positive sentiment lexicon, and words with lowest 100 valence values for the negative sentiment lexicon. The term size in the word cloud is determined by the arousal values of the word. As shown in Fig 11, words can affect the polarity of the reviews. The green words are used for positive movie reviews, and the red words for negative ones. We can see words in the green group, such as 天籟 (heavenly singing voice) [V:7.12, A:5.24] and 完美 (perfect) [V:7.20, A:6.17], indicate that they can trigger positive emotions in the viewer, whereas red words like 絕望 (despair) [V:2.60, A:6.60] and 殘酷 (cruel) [V:2.53, A:8.17] mean that the author has negative feelings towards the movie. For instance, from our movie review corpus is the sentence “聽著梁小琪勇於面對真實感情的天籟, 把氣氛帶向最高潮的完美橋段 (Liang Xiaoqi's heavenly singing voice, which represents the courage to face her true feelings, brings the atmosphere to a perfect bridge to the climax)”; the terms 天籟 (heavenly singing voice) and 完美 (perfect) serve as a complement to describe emotion in their respective phrases. This example shows that the sentiment lexicon generated by calculating the valence-arousal of words can offer great assistance in judging the sentiment polarity of movie reviews. The fusion of valence-arousal information for text representation is effective in sentiment classification. Further, we observed that movie genres such as comedies, romances, and action movies are more likely to induce positive sentiments in viewers, whereas sci-fi, thriller, and horror films often induce negative feelings. Interestingly, the induced sentiments for dramas are almost equally split.

## Concluding remarks

This paper proposes a novel approach to sentiment analysis research, which aims at predicting trends in public opinions using sophisticated methods of emotion analysis. Specifically, we developed a vector-based representation of valence-arousal information that can effectively predict the emotional content of Chinese vocabulary. Using data from the DSA_W competition, the proposed method is shown to achieve the best MAE and impressive PCC performances. In this way, the affective states of a word can be represented as continuous numerical values on multiple dimensions, which enables a fine-grained sentiment analysis. We further developed a Refined Distributed Emotion Vector (RDEV) representation method which is a novel form of text representation that fuses the valence and arousal information behind the text. We can thereby extract emotion vocabulary in the text more efficiently, which leads to improved performances over existing sentiment analysis algorithms. We validated the effectiveness of this model by analyzing film reviews in social media and confirmed that the opinion analysis model based on multi-dimensional emotion information can successfully capture the emotions behind the words used by the authors. In other words, we can accurately analyze the core concepts of the article and the opinion it conveys.

However, this study is not without limitations. Our current model was constructed without the ability to handle negation words; the performance may also be affected due to the neglect of large discrepancies in valence values of the closest words. In light of this, we will investigate the problem of identifying the scope of negation while determining the valence-arousal of a word in the future work. Moreover, we intend to apply this method to English multi-dimensional sentiment and opinion analysis. In addition to film reviews, we will conduct experiments on product reviews or public opinions and expect to achieve similar results so as to validate the generalizability of this method. We will also explore various potential effects of Chinese grammar on valence-arousal predictions, such as negation words, adjectives, and adverbs, as well as word collocations and their interaction on the valence-arousal plane. Finally, we will extend the scope of valence and arousal value prediction to the sentence level.
